# CD28 Co-Stimulus Achieves Superior CAR T Cell Effector Function against Solid Tumors Than 4-1BB Co-Stimulus

**DOI:** 10.3390/cancers13051050

**Published:** 2021-03-02

**Authors:** Ana Textor, Laura Grunewald, Kathleen Anders, Anika Klaus, Silke Schwiebert, Annika Winkler, Maria Stecklum, Jana Rolff, Anton G. Henssen, Uta E. Höpken, Angelika Eggert, Johannes H. Schulte, Michael C. Jensen, Thomas Blankenstein, Annette Künkele

**Affiliations:** 1 Max-Delbrück-Center for Molecular Medicine (MDC), Robert-Rössle-Straße 10, 13092 Berlin, Germany; A.Textor@lumc.nl (A.T.); uhoepken@mdc-berlin.de (U.E.H.); tblanke@mdc-berlin.de (T.B.); 2 Institute of Immunology, Charité Campus Buch, 13092 Berlin, Germany; 3 Department of Hematology, Leiden University Medical Center, 2300 RC Leiden, The Netherlands; 4 Department of Pediatric Oncology and Hematology, Charité—Universitätsmedizin Berlin, Corporate Member of Freie Universität Berlin, Humboldt-Universität zu Berlin, and Berlin Institute of Health, 13353 Berlin, Germany; laura.grunewald@charite.de (L.G.); kathleen.anders@charite.de (K.A.); anika.klaus@charite.de (A.K.); silke.schwiebert@charite.de (S.S.); annika.winkler@charite.de (A.W.); anton.henssen@charite.de (A.G.H.); angelika.eggert@charite.de (A.E.); johannes.schulte@charite.de (J.H.S.); 5 German Cancer Consortium (DKTK), Partner Site Berlin, CCCC (Campus Mitte), 10178 Berlin, Germany; 6 Experimental Pharmacology & Oncology Berlin Buch, 13125 Berlin, Germany; Maria.stecklum@epo-berlin.com (M.S.); ja.ro@web.de (J.R.); 7 Experimental and Clinical Research Center, Lindenberger Weg 80, 13125 Berlin, Germany; 8 Berlin Institute of Health (BIH), 10178 Berlin, Germany; 9 German Cancer Research Center (DKFZ), 69120 Heidelberg, Germany; 10 Seattle Children’s Therapeutics, Seattle Children’s Research Institute, Seattle, WA 98101, USA; michael.jensen@seattlechildrens.org; 11 Fred Hutchinson Cancer Research Center, Seattle, WA 98109, USA; 12 Department of Bioengineering, University of Washington, Seattle, WA 98195, USA

**Keywords:** CAR design, CAR T cell trafficking, preclinical mouse models, neuroblastoma

## Abstract

**Simple Summary:**

Efficient trafficking and survival of CAR T cells within the hostile tumor microenvironment are important prerequisites for potent solid tumor attack that have not yet been achieved. We deployed monospecific murine instead of polyclonal human T cells for CAR T cell generation to evaluate second generation L1CAM- and HER2-specific CARs with different spacer length and either the CD28 or 4-1BB co-stimulatory domain in mouse models of neuroblastoma and ovarian carcinoma. This mouse-in-mouse approach ensured CAR T cell trafficking unhindered by species-specific discrepancies and demonstrated superior solid tumor attack by CAR T cells harboring the CD28 compared to 4-1BB co-stimulatory domain. Our approach has the potential to improve prediction and selection of promising clinical CAR candidates against solid tumors in the future.

**Abstract:**

Spacer or co-stimulatory components in chimeric antigen receptor (CAR) design influence CAR T cell effector function. Few preclinical mouse models optimally support CAR candidate pre-selection for clinical development. Here we use a model in which murine CAR T cells can be exploited with human tumor xenografts. This mouse-in-mouse approach avoids limitations caused by species-specific factors crucial for CAR T cell survival, trafficking and function. We compared trafficking, expansion and tumor control for T cells expressing different CAR construct designs targeting two antigens (L1CAM or HER2), structurally identical except for spacer (long or short) or co-stimulatory (4-1BB or CD28) domains to be evaluated. Using monoclonal, murine-derived L1CAM-specific CAR T cells in Rag-/- mice harboring established xenografted tumors from a human neuroblastoma cell line revealed a clear superiority in CAR T cell trafficking using CD28 co-stimulation. L1CAM-targeting short spacer-CD28/ζ CAR T cells expanded the most at the tumor site and induced initial tumor regression. Treating patient-derived neuroblastoma xenografts with human L1CAM-targeting CAR T cells confirmed the superiority of CD28 co-stimulus. CD28 superiority was also demonstrated with HER2-specific CAR T cells (targeting ovarian carcinoma xenografts). Our findings encourage incorporating CD28 signaling into CAR design for adoptive T cell treatment of solid tumors.

## 1. Introduction

Chimeric antigen receptor (CAR) T cell therapy is a promising clinical approach in cancer treatment where patients’ own T cells are engineered to express a synthetic receptor that redirects specificity towards recognizing and killing cancer cells. First-generation CAR design contained an extracellular antibody-derived antigen-binding domain (scFv) and a single intracellular CD3ζ signaling domain connected via an extracellular spacer and transmembrane domain. Due to limited efficacy in clinical trials, one (second generation) or two (third generation) co-stimulatory signaling domains (most often CD28 or/and 4-1BB) were added to improve CAR T cell persistence and, hence, effector functions [1,2,3]. While CARs implementing CD28 co-stimulation demonstrated brisk and strong effector function, 4-1BB signaling resulted in slower but more durable T cell responses [4]. Here, we use CAR T cells targeting the glycosylated CE7 epitope of the L1 cell adhesion molecule, L1CAM (formerly CD171), which is specifically expressed on tumor cells and a promising target for neuroblastoma and ovarian cancer [5,6,7]. Neuroblastoma is the most common extracranial solid tumor in childhood and remains the third leading cause of pediatric cancer death despite multimodal therapies [8,9]. Recently, children diagnosed with refractory neuroblastoma were treated with L1CAM-targeting CAR T cells harboring the 4-1BB co-stimulatory domain in a clinical phase I trial (NCT02311621, Available online: https:clinicaltrials.gov (accessed on 1 March 2021)).

Knowing that minor differences in CAR design, such as spacer length, can significantly impact CAR T cell functionality [10,11] led to extensive preclinical testing of L1CAM-specific CAR T cells harboring a short, medium or long spacer element. While CAR T cells harboring the long spacer were superior in vitro, they failed in vivo due to activation-induced cell death [5]. A preclinical NOD *scid* gamma mouse model using the human SK-N-BE(2) neuroblastoma cell line, human L1CAM-specific CAR T cells and intratumoral T cell injection revealed superior effector function of the short spacer CAR T cells. Based on these results, the short spacer-4-1BB CAR design was selected for testing in the above-mentioned clinical trial. However, this mouse model has critical limitations. Firstly, human T cells transferred into a mouse environment lack an unknown number of growth and survival factors, limiting survival and engraftment capacities. Secondly, chemokine and adhesion molecule activity could be species-specific, impeding efficient trafficking and extravasation of human T cells in a murine host [12,13]. Intratumoral injection is often used to circumvent this limitation making it impossible to identify CAR T cell designs conferring superior homing capacities. Thirdly, human T cell-secreted interferon gamma (IFNG), which is known to be important for tumor eradication, does not act on murine tumor stromal cells [14,15]. Finally, using human T cells in preclinical mouse models will eventually induce graft-versus-host disease because they are xenogenic to the host. This limits the time window for observation of anti-cancer effects, which especially in solid tumor treatment could be delayed.

Here we investigated whether CAR T cells harboring the CD28 instead of 4-1BB co-stimulatory domain possess stronger effector function, making them advantageous for solid tumor treatment. We developed a new mouse model for this purpose. Using monoclonal murine instead of polyclonal human T cells for CAR T cell generation ensured appropriate responsiveness of CAR T cells to murine factors important for trafficking and enabled assessment of homing capacity in different CAR T cell designs.

## 2. Materials and Methods

### 2.1. Cell Lines and Culture Conditions

SK-N-BE(2) (neuroblastoma), SKOV3 (ovarian carcinoma) and 293T (lentiviral vector packaging cell line) were cultured in Dulbecco’s Modified Eagle Medium (DMEM; Life Technologies) supplemented with 10% heat-inactivated fetal calf serum (FCS; Sigma). Cell lines for this study were authenticated by Eurofins and *Mycoplasma*-negatively tested by a cell-based colometric HEK-Blue Detection assay (Invivogen, San Diego, CA, USA).

### 2.2. CAR Construct Generation

For transduction of human T cells, the previously described L1CAM-specific CE7-CAR [16] was cloned into the SIN epHIV7 lentiviral vector. Single-chain variable fragments in the L1CAM- and HER2-specific CAR constructs were codon-optimized and subsequently linked to a spacer domain from the human IgG4-Fc hinge with 12 (short) or 229 (long) amino acids. The long spacer was modified by substituting L235D and N297Q [17,18]. The spacer domain connects the antigen-binding domain to the CD28 transmembrane domain followed by the signaling module containing either the 4-1BB or CD28 co-stimulatory domain and the CD3ζ cytoplasmic domain. CAR constructs were linked downstream to a T2A self-cleaving peptide and a truncated epidermal growth factor receptor (EGFRt) allowing CAR T cell detection and enrichment [19]. For murine T cell transduction, L1CAM- and HER2-specific CAR constructs were introduced into the MP71 [20] gamma-retroviral vector.

### 2.3. Gamma-Retroviral and Lentiviral Vector Production, Transduction and CAR T Cell Generation

To generate gamma-retroviral supernatants, 293T cells were co-transfected with MP71-CAR constructs and gag, pol and env encoding pCL-eco vector (Imgenex) as previously described [15]. Virus-containing supernatants were collected 48 and 72 h post-transfection, filtered (0.45 µm pore size) and either used directly for transduction or stored at −80 °C. Murine CAR T cells were generated and cultured as previously described, but using 40 IU/mL IL2 [21]. CAR-encoding lentiviral supernatants were produced via transient 293T cell transfection as previously described [22]. Briefly, 293T cells were transfected with the SIN epHIV7 lentiviral plasmid encoding the CAR and three additional plasmids encoding for *gag*/*pol* gene, *rev* gene and *VSV-G* gene using the CalPhos-Mammilian Transfection kit (Takara). Supernatants were collected 48 and 72 h post-transfection and concentrated by ultracentrifugation. Human CAR T cells were generated from healthy donors (Charité ethics committee approval EA2/262/20) and cultured as previously described [16].

### 2.4. Flow Cytometry

Cell surface expression of L1CAM (clone REA163, Miltenyi Biotec, Bergisch Gladbach, Germany), GD2 (clone 14.G2a; BD), HER2 (clone 24D2, Biolegend, San Diego, CA, USA) and CD8 (clone SK1, BioLegend) was detected by fluorophore-conjugated monoclonal antibodies. EGFRt expression was detected using biotinylated cetuximab (Bristol-Myers Squibb) and a phycoerythrin (PE)-conjugated streptavidin antibody (cat #12-4317-87, BioLegend). The APC Annexin V Apoptosis detection kit (cat #422201, BioLegend) was used to assess apoptosis after 24, 48 and 72 h of stimulation with L1CAM^+^ tumor cells (E:T ratio, 1:5). Dead cells were excluded from analyses using LIVE/DEAD^TM^ Fixable Green Dead Cell Stain Kit (cat #L23101, Life Technologies). Cell surface expression from murine T cells was identified using Cd8 (clone 53-6.7, BD Bioscience, San Jose, CA, USA), Pd1 (clone J44, BD Bioscience) and Tim3 (clone RMT3-23, BD Bioscience). Flow cytometry was performed on Fortessa X-20 (BD Biosciences) and FACSCanto II (BD Biosciences) instruments. Data was processed using FlowJo_V10 Software (Tree Star Inc., Ashland, OR, USA).

### 2.5. Bioluminescence-Based Cytotoxicity and Cytokine Release Assays

CAR T cell-induced cytotoxicity was quantified in a bio-photonic luciferase assay in which neuroblastoma cells stably transduced with a GFP-ffLuc_epHIV7 reporter served as tumor target cells. Target cells were co-cultured for 24, 48 or 72 h in triplicate with CAR or untransduced T cells. Tumor cells were quantified by bioluminescent signal quantification on a GloMax 96 Microplate Luminometer (Promega) after adding 0.14 mg/mL d-luciferin (PerkinElmer Inc., Waltham, WA, USA) to each well. Lysis was determined as [1 − (RLUsample/RLUmax)] × 100 in relation to untreated cells. For cytokine release assays with human CAR T cells, 1 × 10^5^ T cells were seeded into wells (24-well plates) together with stimulator cells at a 1:5 effector:target ratio. E:T ratios were titrated by seeding 1 × 10^5^ transduced murine CAR T cells with diluted numbers of tumor cells (5 − 5 × 10^4^). All data points were technical duplicates or triplicates. Conditioned media was collected after 24 h and stored at −80 °C until IFNG/Ifng and IL2/Il2 analysis using human or mouse OptEIA™ Set (BD Biosciences) enzyme-linked immunosorbent assay (ELISA) kits according to the manufacturer’s instructions.

### 2.6. In Vivo Studies

Animal experiments were conducted in accordance with institutional and national guidelines and regulations after Landesamt für Gesundheit und Soziales (LAGeSo, Berlin, Germany) approval. Albino Rag1^−/−^ or Rag2^−/−^ (Rag^−/−^), ChRLuc/OT-1/Rag1^−/−^ mice were previously described [23]. For experiments in Rag^−/−^ mice, 5 × 10^6^ SK-N-BE(2) cells in 50% Matrigel™/50% phosphate-buffered saline (PBS) or 5 × 10^6^ SKOV3 cells in 100 μL PBS were subcutaneously injected into age- and sex-matched mice. Mice were ranked by tumor size on the day of CAR T cell treatment, and treatment groups were randomized and contained mice with similar mean tumor sizes. Mice received intravenous injection of 1 × 10^6^ CAR T cells or untransduced T cells in 100 μL PBS. Tumor size was measured by an electronic caliper along three orthogonal axes (a, b and c), and tumor volumes were calculated by V(mm^3^) = (a × b × c)/2. Mice were sacrificed when tumors reached 15 mm mean diameter. CAR T cell trafficking and expansion in vivo was followed using bioluminescent imaging on a Xenogen IVIS 200 (Caliper Lifescience, Hopkinton, MA, USA) as previously described [23]. Data were analyzed using Living Image software (Caliper Lifescience). EPO Berlin-Buch GmbH conducted experiments in NOG mice harboring patient-derived neuroblastoma xenografts using LAGeSo-approved protocols. Patient-derived xenografts (PDXs) were subcutaneously transplanted into the flank of NOG mice and passaged by removing tumors, mincing tumor material and re-transplanting it into mice. Female mice between 6–8 weeks of age and PDXs with five to eight passages were used for blinded experiments. Animals received 1 × 10^7^ human L1CAM-specific CAR T cells intravenously on three consecutive days. Tumor growth was measured bi-weekly and quantified by caliper measurement.

### 2.7. Tumor-Infiltrating T Cell (TIL) Isolation

For TIL isolation, tumors were harvested, halved, weighed, sliced and incubated for 1 h at 37 °C in 10 mL complete RPMI 1640 medium with collagenase II (1 mg/mL, Gibco, Billings, MT, USA), Dispase II (1 mg/mL, Roche, Basel, Switzerland), and DNAse I (10 μg/mL, Roche). Tumor cells were dissociated by passing through mesh filters (40 μm), washing with PBS and treating with ammonium-chloride-potassium lysing buffer. CD8 T cells were purified from tumor cell suspension by using anti-CD8 monoclonal antibody microbeads (Miltenyi Biotec, Bergisch Gladbach, Germany) according to the manufacturer’s protocol. Purified cells were counted and the CD8^+^ T cell percentage within the purified cell fraction was flow cytometrically determined.

### 2.8. Statistical Analysis

Significant differences in cytotoxic activity, cytokine release and apoptosis in CAR T cells compared with untransduced T cells were determined in paired and unpaired Student’s *t*-tests. TIL numbers were compared using one-way ANOVA. Mouse cohorts treated with L1CAM-specific CAR T cells or untransduced T cells were compared using Kaplan–Meier survival analysis with log-rank statistics. All analyses were performed using GraphPad prism (GraphPad Software version 8.0). *p* values < 0.05 were considered statistically significant.

## 3. Results

### 3.1. Short Spacer L1CAM-Specific CAR T Cells with CD28 Signaling Prolonged Survival of SK-N-BE(2) Tumor-Bearing Mice

**Figure 1:**
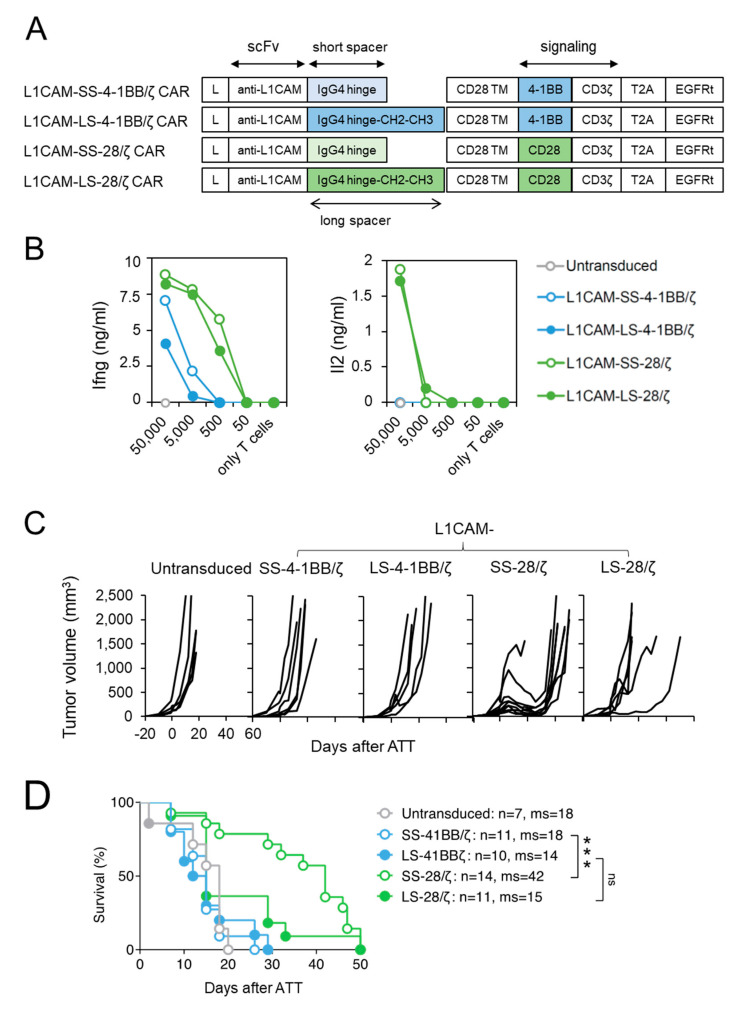
Murine CD8^+^ T cells expressing short spacer L1CAM-specific CARs with CD28/ζ signaling domain show superior anti-tumor activity in vivo. (**A**) Scheme of retroviral CAR T cell constructs used to generate L1CAM-specific second-generation CAR T cells harboring a short or long spacer with 4-1BB or CD28 signaling domain. L: leader sequence; TM: transmembrane domain; T2A: virus 2A self-cleaving sequence, EGFRt, truncated epidermal growth factor receptor. (**B**) CD8^+^ T cells derived from ChRLuc/OT-1/Rag^−/−^ transduced with indicated CAR constructs were co-cultured with titrated numbers of SK-N-BE(2) target cells for 24 h. Levels of secreted cytokines were measured by Ifng and Il2 ELISA. Shown is one representative experiment of three. (**C**) Tumor growth curves of SK-N-BE(2) tumor-bearing Rag^−/−^ mice treated with 1 × 10^6^ EGFRt^+^ ChRLuc/OT-1/Rag^−/−^ CAR T cells transduced with L1CAM-SS-4-1BB/ζ (*n* = 6), L1CAM-LS-4-1BB/ζ (*n* = 5), L1CAM-SS-28/ζ (*n* = 9), L1CAM-LS-28/ζ (*n* = 6) or with untransduced T cells (*n* = 5) at indicated time-point. Each line represents change in tumor volume of an individual mouse over the period of the experiment. Data are representative of two independently performed experiments. (**D**) Kaplan–Meier survival analysis is shown from total mice for each indicated CAR T cell treatment. Combined data from two experiments are shown. Statistical significances are indicated as ***, *p* ≤ 0.005. ns = not statistically significant, SS = short spacer, LS = long spacer, ATT = adoptive T cell transfer, ms = median survival.

In a previous study, we compared human second generation L1CAM-secific CAR T cell designs harboring a 4-1BB co-stimulatory domain with either a short, intermediate or long spacer element and demonstrated superior in vivo function of CARs with the short spacer [15]. To extend our findings for anti-cancer potential beyond the effect of spacer domain length [16], we generated L1CAM-specific CAR designs with CD28-costimulatory signaling incorporating a short (L1CAM-SS-CD28/ζ) or long (L1CAM-LS-CD28/ζ) spacer, and compared their anti-tumor activity to short (L1CAM-SS-4-1BB/ζ) and long spacer (L1CAM-SS-4-1BB/ζ) murine CAR T cells with 4-1BB-co-stimulatory signaling (Figure 1A). The vector also included a truncated EGF receptor (EGFRt), used as a surrogate marker to assess transduction efficacy. Transduction of murine T cells was successful for all constructs (Supplementary Figure S1A). Murine CAR T cells were assessed in vitro to investigate CAR construct functionality, since components except for the scFv were human-derived. After co-culture of murine L1CAM-specific CAR T cells with titrated numbers of L1CAM^+^ SK-N-BE(2) neuroblastoma cells (Supplementary Figure S2A), Ifng and Il2 cytokine release were quantified by ELISA (Figure 1B). All L1CAM-specific constructs induced Ifng release in T cells upon antigen encounter, but less Ifng was secreted by T cells with 4-1BB signaling. Neuroblastoma cells induced Il2 release only in CAR T cells utilizing CD28 signaling. Here, we demonstrate that murine T cells equipped with L1CAM-specific CAR constructs harboring human-derived signaling domains efficiently produce cytokines after encounter of L1CAM antigen on human neuroblastoma cells.

To assess anti-tumor activity in vivo, we performed adoptive transfer experiments in Rag^−/−^ mice with established SK-N-BE(2) neuroblastoma xenografts implanted subcutaneously and grown for 20 days. Murine T cells were intravenously injected to evaluate their capacity to home to and infiltrate the tumor. Murine T cells were used to ensure appropriate function of host factors critical for T cell trafficking (e.g., chemokines) and tumor stroma targeting (e.g., Ifng). To avoid xenoreactivity of polyclonal murine T cells towards human neuroblastoma xenografts and to be able to follow T cell homing in vivo by bioluminescent imaging, CARs were introduced into CD8^+^ T cells derived from ChRLuc/OT-1/Rag^−/−^ transgenic mice that exclusively express an OVA-specific tumor-unrelated TCR and are transgenic for *Renilla* luciferase (RLuc) [23]. Similar to mice receiving untransduced T cells, no SK-N-BE(2) tumor regression occurred in mice intravenously injected with 1 × 10^6^ CD8^+^ mouse CAR T cells, expressing either the short or long spacer L1CAM-specific 4-1BB/ζ CARs (Figure 1C). In contrast, L1CAM-SS-28/ζ-CAR T cells induced tumor regression in eight of nine SK-N-BE(2) tumor-engrafted mice and significantly increased mean overall survival (Figure 1C). L1CAM-LS-28/ζ CAR T cells delayed tumor growth in one of six mice, demonstrating no significant improvement in overall survival (Figure 1C,D). Taken together, CD28 co-stimulus was superior for inducing regression of established neuroblastoma xenografts by L1CAM-specific mouse CAR T cells, predominantly those harboring the short spacer domain.

### 3.2. Anti-Tumor Effect of L1CAM-Specific CAR T Cells Correlates with T Cell Expansion at the Tumor Site

**Figure 2:**
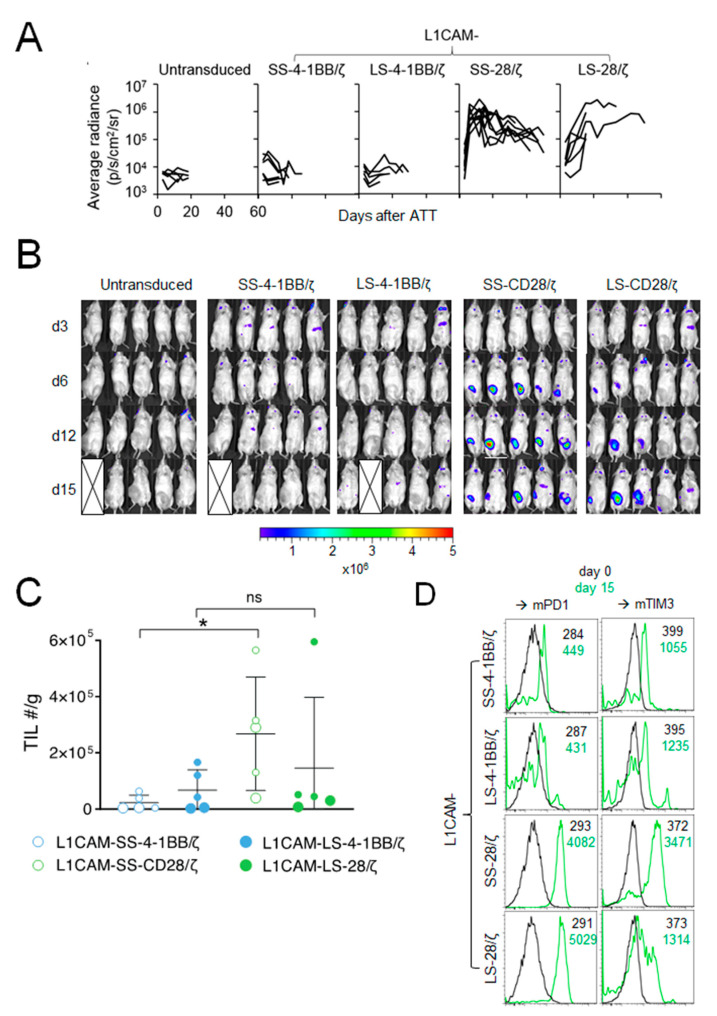
The CD28 signaling domain enables L1CAM-specific CAR T cells with short and long spacer to expand at the tumor site. (**A**) Kinetic of the T cell signal (average radiance) detected at the tumor site over time is shown for mice from Figure 1C (L1CAM-SS-4-1BB/ζ (*n* = 6), L1CAM-LS-4-1BB/ζ (*n* = 5), L1CAM-SS-28/ζ (*n* = 9), L1CAM-LS-28/ζ (*n* = 6), untransduced (*n* = 5)). (**B**) Bioluminescent imaging data of mice shown in (**A**) on indicated time points following treatment (5 representative mice are shown for each group). The pseudo color scale indicates the intensity of the signal and is set from minimum 2 × 10^5^ to maximum 5 × 10^6^ p/s/cm^2^/sr. Data are representative of two independently performed experiments. (**C**) Mice were treated as described in (**A**) and were sacrificed on day 15. Tumors were harvested, T cells extracted and quantified. Total numbers (#) of TILs/g of tumors are depicted. Larger circles represent TILs of mice analyzed in (**D**). (**D**) TILs isolated from two mice of each treatment group shown in (**C**) were analyzed by flow cytometry for Pd1^+^ and Tim3^+^ expression and expression levels were compared to CAR T cells before infusion (d0). Black and green numbers within the graphs present mean fluorescent intensity (MFI) values of T cells before infusion and TILs isolated on day 15, respectively. Error bars represent SD, ns = not statistically significant *, *p* ≤ 0.05; TIL = tumor infiltrating lymphocytes.

To elucidate why L1CAM-specific CAR T cells harboring the 4-1BB co-stimulatory domain performed poorly in vivo compared to CAR T cells with CD28 co-stimulation, we studied homing capacity of RLuc-transgenic T cells harboring the different L1CAM-specific CAR constructs. None of the SK-N-BE(2) tumor-engrafted mice intravenously treated with 1 × 10^6^ L1CAM-SS-4-1BB/ζ or L1CAM-LS-4-1BB/ζ CD8^+^ murine T cells showed increased bioluminescence at the tumor site (Figure 2A,B). In contrast, bioluminescence at the tumor site rapidly increased in all mice following L1CAM-SS-28/ζ CAR T cell injection, demonstrating efficient CAR T cell homing and expansion (Figure 2A,B). L1CAM-LS-28/ζ CAR T cells produced a mixed response, since bioluminescence at the tumor site increased only in some mice (Figure 2B). The bioluminescence increase was also delayed by 1 week compared to L1CAM-SS-28/ζ CAR T cells, indicating less efficient CAR T cell homing or expansion at the tumor site. To go beyond the sensitivity of bioluminescent imaging and investigate phenotypic differences between tumor-infiltrating T cells (TILs) from the different treatment groups, we treated a new cohort of SK-N-BE(2) tumor-bearing mice with different L1CAM-specific CAR T cell subsets. Tumors were harvested 15 days after T cell infusion for TIL quantification and exhaustion marker analysis. While TILs were detected in all treatment groups, indicating that T cells of all subgroups survived, T cell infiltration was highest in tumors treated with L1CAM-SS-28/ζ CAR T cells (Figure 2C). A similar degree of T cell infiltration occurred only in one of five mice treated with L1CAM-LS-28/ζ CAR T cells (Figure 2C). Tenfold lower CAR T cell infiltration was detected in all mice treated with L1CAM-LS-28/ζ or 4-1BB co-stimulus containing CARs, confirming our results obtained by bioluminescent imaging. Although L1CAM-SS-28/ζ CAR T cells demonstrated superior anti-tumor activity in vivo, tumor control was not persistent and tumors in all mice eventually relapsed. A lack of durable anti-cancer activity can be caused by CAR T cell exhaustion, which is preferentially observed in CAR T cells with CD28 co-stimulation after repeated antigen-encounter [11]. Indeed, TILs in both, L1CAM-SS-28/ζ- and L1CAM-LS-28/ζ-treated mice expressed higher levels of PD-1 and T cell membrane protein 3 (TIM-3) 15 days after injection as compared to CAR T cells before infusion or as seen in 4-1BB harboring CAR T cells (Figure 2D). These data suggest all CAR T cell subsets trafficked to the tumor site, but once there, differed in their ability to expand and remain functional.

### 3.3. Superior Function of Human L1CAM-SS-28/ζ CAR T Cells Is Confirmed in a PDX Mouse Model

**Figure 3:**
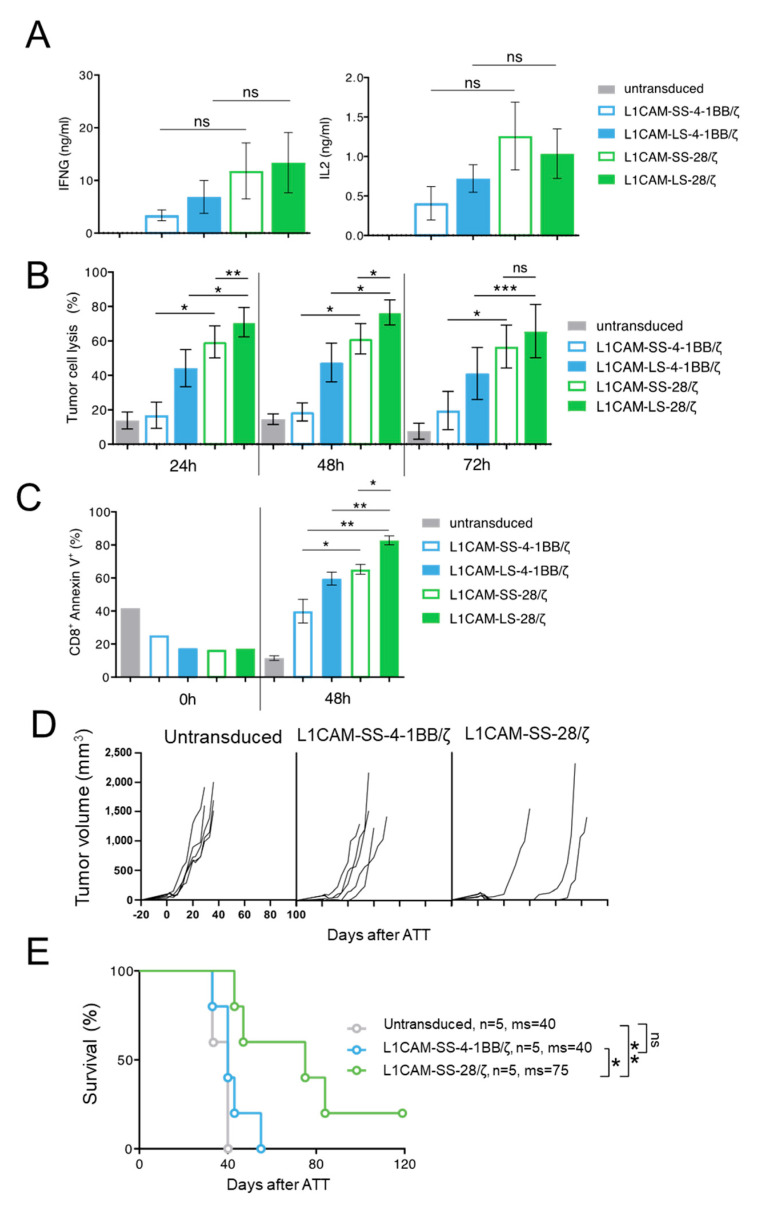
The superior function of CD28-based CAR T cells can be confirmed in a PDX mouse model using human T cells. (**A**) IFNG and IL2 cytokine secretion after 24 h co-culture of untransduced T cells or different L1CAM-specific CAR T cell constructs with SK-N-BE(2) target cells in an effector to target (E:T) ratio of 1:5 assessed by ELISA (*n* = 3); Error bars represent SD. (**B**) Cytolytic activity of the four L1CAM-specific CAR T cell subgroups against SK-N-BE(2) neuroblastoma cells determined by luciferase-based killing assay following a co-culture for 24, 48 and 72 h at an E:T ratio of 1:5. Data shown here, depict the mean cytolytic activity of 3 independent experiments. Error bars represent SD. (**C**) Quantification of apoptosis of CAR T cells by annexin V staining before and after co-culture with SK-N-BE(2) cells for 48h in an E:T of 1:5. by two tailed paired *t* test. (**D**) NOG mice with subcutaneous neuroblastoma PDXs were treated by intravenous injection of 1 × 10^7^ L1CAM-specific SS-4-1BB/ζ (*n* = 5), SS-28/ζ (*n* = 5) CAR or untransduced (*n* = 5) T cells on three consecutive days. Each line represents change in tumor volume of an individual mouse over the period of the experiment. (**E**) Kaplan–Meier survival analysis of mice shown in (**D**). ns = not statistically significant; ms = median survival, *, *p* ≤ 0.05; **, *p* ≤ 0.01; ***, *p* ≤ 0.005, Kaplan–Meier survival analysis with log-rank statistics.

To test if our findings are translatable from murine to human CAR T cells, we transduced human CD8^+^ T cells with the four L1CAM-specific CAR constructs and enriched transduced T cells by selection for EGFRt^+^ expression using magnetic-activated cell sorting (MACS) (Supplementary Figure S1B). To investigate human CAR T cell effector function in vitro, SK-N-BE(2) neuroblastoma cells were co-cultured with different L1CAM-specific CAR T cell subsets or untransduced T cells. Although differences in IFNG and IL2 cytokine release were not significant after 24 h of co-culture, neuroblastoma cell encounter induced higher IFNG and IL2 release from human L1CAM-specific CAR T cells harboring the CD28 instead of 4-1BB co-stimulatory domain (Figure 3A), following the trend observed in murine CAR T cells. Differing from what we observed in murine T cells, both CAR T cells with 4-1BB co-stimulation produced IL2, but in lower amounts than both CAR T cells with CD28 co-stimulation. We assessed cytotoxic potential of the four human L1CAM-specific CAR T cell subsets using a luciferase-based reporter assay. CAR T cell subsets or untransduced T cells were co-cultured with transgenic SK-N-BE(2) neuroblastoma cells expressing firefly luciferase 24, 48 and 72 h before measuring the bio-photonic signal released by remaining viable tumor cells (Figure 3B). Human L1CAM-specific CAR T cells harboring short- or long-spacer CAR constructs with CD28 co-stimulation lysed significantly more tumor cells than CAR T cells using 4-1BB signaling at each time point analyzed. Interestingly, L1CAM-LS-28/ζ CAR T cells demonstrated a significantly stronger killing ability than L1CAM-SS-28/ζ CAR T cells after 24 h of co-culture. This result stands in contrast to anti-cancer efficacy observed with murine CAR T cells in vivo. To assess why superior in vitro performance of L1CAM-LS-28/ζ CAR T cells was not translated into superior anti-cancer activity in vivo, we monitored whether CAR T cell survival differed between the individual CAR T cell subsets after antigen exposure. After co-culturing human L1CAM-specific CAR T cells with SK-N-BE(2) cells for 48h, we quantified the apoptotic CAR T cell fraction by annexin V-labeling (detecting cell surface phosphatidylserine exposure, a marker for apoptotic cell death). Gating CD8^+^/GD2^−^/annexin V^+^ cells (Supplementary Figure S3) presented larger apoptotic fractions in L1CAM-LS-28/ζ CAR T cells than all other CAR T cell subsets (Figure 3C), indicating that L1CAM-LS-28/ζ CAR T cells are more prone to undergo activation-induced cell death after antigen encounter. Comparable to what we have observed for murine CAR T cells, CD28 co-stimulus induced greater effector function in human CAR T cells in vitro compared to T cells with 4-1BB co-stimulus, but L1CAM-LS-28/ζ CAR T cells are more likely to undergo activation-induced cell death.

After having shown that murine CAR T cells equipped with L1CAM-specific CAR constructs utilizing CD28 co-stimulation performed better in vivo, we wanted to analyze whether human L1CAM-SS-28/ζ CAR T cells were also more potent in vivo than their 4-1BB counterparts. For a clinically relevant setting, we chose a neuroblastoma PDX mouse model with high L1CAM expression on the tumor cells (Supplementary Figure S2B). Tumor-bearing NOG mice were treated with human L1CAM-SS-28/ζ CAR T cells, whose murine counterpart performed best in our SK-N-BE(2) mouse model and compared anti-cancer efficacy to L1CAM-SS-4-1BB/ζ CAR T cells. Treating our Rag^−/−^ model with similarly sized tumors (200–250 mm^3^) with the same dose of human L1CAM-specific CAR T cells (1 × 10^6^) produced no anti-tumor effect. Tumors in both CAR T cell treatment groups were no different to mice treated with untransduced T cells (Supplementary Figure S4). The attempt to detect CAR T cells in peripheral blood (9 and 15 day after CAR T cell infusion) or in the harvested tumors using antibodies to human CD45 remained negative (data not shown), indicating poor in vivo human T cell survival in mice. When we administered tenfold (1 × 10^7^) human L1CAM-specific CAR T cell doses on three consecutive days and started treatment earlier when tumors became just palpable (mean diameter 30–100 mm^3^), L1CAM-SS-28/ζ CAR T cells were able to eradicate the tumor in only 1 of 4 mice without recurrence during the observation period of >100 days (Figure 3D). Although the fifth mouse in this cohort needed to be sacrificed due to discomfort not caused by the tumor on day 60, survival was significantly improved by treatment (Figure 3E). These results confirmed that CD28 co-stimulation, in a clinically relevant PDX mouse model, also provided a more potent in vivo anti-tumor effect in human L1CAM-specific CAR T cells.

### 3.4. HER2-Specific CAR T Cells with CD28 Co-Stimulation Possess Higher Effector Function

**Figure 4:**
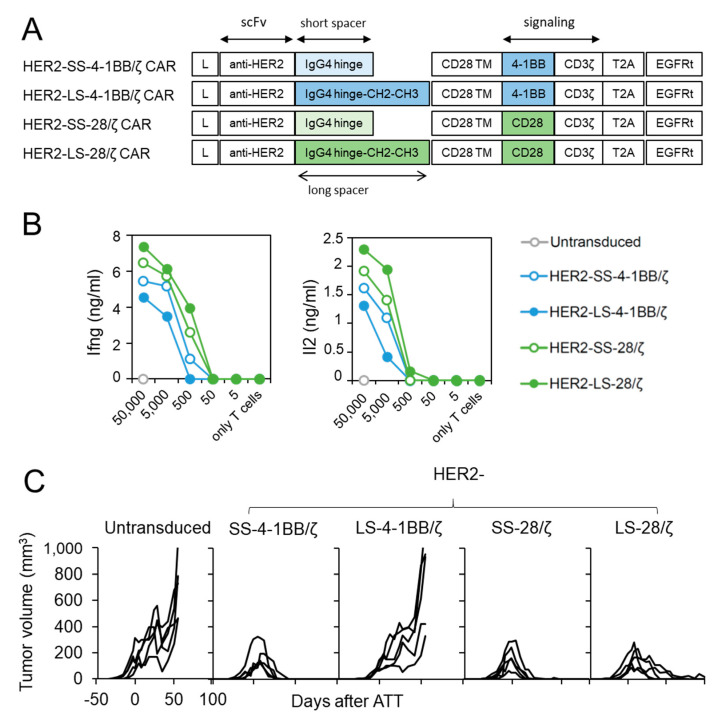
HER2-specific CAR T cells with CD28 signaling outperform 4-1BB harboring CAR T cells. (**A**) Scheme of retroviral CAR T cell constructs used to generate HER2-specific second-generation CAR T cells harboring a short or long spacer with 4-1BB or CD28 signaling domain. L: leader sequence; TM: transmembrane domain; T2A: virus 2A self-cleaving sequence, EGFRt, truncated epidermal growth factor receptor. (**B**) CD8^+^ T cells derived from ChRLuc/OT-1/Rag^−/−^ transduced with indicated CAR constructs were co-cultured with titrated numbers of SKOV3 target cells for 24 h. Levels of secreted cytokines were measured by Ifng and Il2 ELISA. Shown is one representative experiment of three. (**C**) Rag^−/−^ mice were subcutaneously injected with SKOV3 tumor cells and treated with 1 × 10^6^ CAR T cells equipped with the indicated HER2-specific CAR constructs or with untransduced T cells (*n* = 5 for each treatment group). Tumor volume was determined by caliper measurement. A single experiment is shown with each line representing change in tumor volume of an individual mouse over time.

We next investigated whether CD28 co-stimulation confers superior anti-cancer activity against another tumor entity and antigen. For this purpose, we generated structural identical CAR T cell constructs with a HER2-specific scFv [15] (Figure 4A) and transduced them into CD8^+^ T cells derived from ChRLuc/OT-1/Rag^−/−^ mice (Supplementary Figure S1C). In vitro encounter with HER2^+^ SKOV3 ovarian carcinoma cells (Supplementary Figure S2C) induced Ifng and Il2 release from all four HER2-specific CAR T cell designs regardless of co-stimulatory moiety, but designs using 4-1BB co-stimulation also induced lower cytokine levels as in CAR T cells targeting L1CAM (Figure 4B). To investigate whether in vivo anti-cancer potential of HER2-specific CAR T cells utilizing 4-1BB or CD28 signaling differ, we treated SKOV3 tumor-bearing Rag^−/−^ mice by a single intravenous injection of untransduced or HER2-specific SS-4-1BB/ζ, LS-4-1BB/ζ-, SS-28/ζ-, LS-28/ζ-CAR CD8^+^ T cells. Both short-spacer CAR T cell subsets eradicated established SKOV3 tumors in all mice with kinetics comparable to CAR T cells targeting L1CAM (Figure 4C). The anti-cancer activity of HER2-specific CAR T cells with long spacers differed, with HER2-LS-28/ζ CAR T cells achieving slightly delayed tumor eradication in all mice and HER2-LS-4-1BB/ζ CAR T cells failing to induce any significant tumor regression. These findings demonstrate that HER2-specific CAR T cells with a short spacer element performed equally well, irrespective of co-stimulatory domain used, and that CAR designs benefit from CD28 co-stimulation in the ovarian tumor model.

## 4. Discussion

CAR T cell therapy has achieved remarkable responses against hematological malignancies, but success remains limited against solid tumors. Novel CAR designs developed to increase CAR T cell persistence, effector response magnitude and safety have the potential to improve CAR T cell-based immunotherapy against solid tumors, but appropriate preclinical mouse models are required to identify the most potent CAR design worth further clinical investigation. Currently, the state-of-the-art mouse model for preclinical CAR T cell evaluation utilizes human-derived T cells against human tumor xenografts. Due to the fact that an unknown number of host (mouse) and T cell (human) factors act in a species-specific manner, important aspects of successful T cell therapy, such as CAR T cell homing to the tumor site, cannot be studied in these models. Destruction of mouse tumor stroma by human T cell effector molecules such as IFNG is also excluded in these models but is critical for solid tumor eradication [15]. A mouse model that can overcome these limitations would be of utmost importance for pre-selecting suitable CAR designs for clinical development.

Here we compared structurally identical CAR T cell designs targeting either L1CAM or HER2, and differing only in the case of two components, space length and type of co-stimulatory domain. We particularly focused on a more complete understanding of the influence of these CAR components on T cell migration and tumoricidal potency in vivo. We used murine instead of human T cells to ensure appropriate responsiveness to murine factors important for T cell trafficking and stroma targeting by T cell effector molecules. Murine T cells were derived from ChRLuc/OT-1/Rag^−/−^ transgenic mice whose T cells are mono-specific against a cancer-unrelated antigen (ovalbumin) to exclude CAR-unrelated T cell responses against the host or the human tumor xenograft and express *Renilla* luciferase to allow non-invasive T cell tracking over time. We showed for both CAR specificities that T cells with the CD28 co-stimulatory endodomain migrated better to the tumor and had superior anti-tumor effect, while T cells with 4-1BB co-stimulus (except HER2-SS-4-1BB/ζ) failed to expand at the tumor site and induce tumor regression. This finding was surprising and stands in contrast to other studies comparing anti-cancer potential of CD28- or 4-1BB-incorporating CAR designs to combat solid tumors. Cherkassky et al. compared CD28- and 4-1BB-incorporating mesothelin-specific second-generation CAR T cells and demonstrated both CAR T cell designs killed orthotopic tumors equally well [24]. Priceman et al. demonstrated superior in vivo function of 4-1BB-incorporating CAR T cells targeting prostate stem cell antigen (PSCA) compared those using the CD28 endodomain [25]. Two factors could account for the observed discrepancy. Firstly, our CAR designs were structurally identical except for two features (spacer length and co-stimulatory domain), while CAR design in the published studies incorporated not only the CD28 and 4-1BB co-stimulatory domains but also their transmembrane domains, which can influence the stability of CAR-derived surface protein expression, hence anti-cancer potential [26]. Secondly, and more importantly, the targeted tumor size most likely differed. We treated tumors of at least 5 mm in diameter that grew for at least three weeks. Tumor burden was determined by bioluminescent signal quantification in both published studies, but actual tumor size at treatment was not specified. It, however, is likely that intrapleurally [24] or intratibially [25] placed tumors could not exceed a few millimeters in diameter before experimental endpoints were reached. In our experience, only tumors allowed to grow to at least 5 mm over a period of several weeks mimicked clinically relevant characteristics of solid tumors, such as an established tumor microenvironment including immunosuppressive stromal cells and a tumor-supplying vasculature. Solid tumor eradication requires simultaneous destruction of the tumor stroma by T cell effector molecules such as IFNG [14,27,28]. We hypothesize that superior T cell expansion combined with the faster and stronger cytokine response characteristic for CD28-incorporating second-generation CAR T cells produces sufficiently high cytokine levels to destroy tumor stroma. If this is the case, the superior function of CD28- compared to 4-1BB-incorporating CAR T cells would only become apparent when tumors with an established tumor stroma are treated. In blood-born cancers, these fundamental differences in responses conveyed by CD28- and 4-1BB-incorporating CAR T cells might not play a significant role, because target cells are immediately accessible, ensuring a strong initial CAR T cell expansion independent of the co-stimulatory domain used and compensating for the slower and reduced effector molecule production by 4-1BB-incorporating second-generation CAR T cell designs.

We confirmed superiority of L1CAM-SS-28/ζ to L1CAM-SS-4-1BB/ζ CAR T cells in experiments treating mice harboring PDX tumors with human CAR T cells, but repeated injections of 10-fold more murine T cells were required to achieve a measurable therapeutic effect. While PDXs might be more difficult to treat per se, an early disappearance of human T cells in the blood of treated mice points to low survival in the xenogenic environment, as does no observation of rapid graft-versus-host disease. To compensate for low persistence, a large percentage of CAR candidates now under clinical investigation were assessed by intratumoral CAR T cell delivery in preclinical mouse models, including CARs against L1CAM [16], HER2 [29], IL13Rα2 [30]. Extreme T cell doses have also been combined with high-dose IL2 treatment to promote human T cell persistence in mice [31,32]. The intravenous CAR T cell delivery in our mouse-in-mouse model better mimics the typical clinical delivery in the patient. In this system, we showed lack of effector function in L1CAM-specific CAR T cells with 4-1BB co-stimulus, one of which was previously selected for a clinical trial because of proven anti-cancer activity after intratumoral delivery. Injection route dramatically impacts CAR T cell efficacy, implying that similar delivery (local versus systemic) should be followed during pre- and clinical testing. In this age of advanced in vitro model development, our findings also highlight that testing in whole-organism models still has its place in preclinical evaluation of CAR T cell candidates, since complex aspects such as T cell migration to and into the tumor or clearance from the bloodstream cannot be fully mimicked in vitro using tumor-building technologies such as organoids.

Although both murine L1CAM-specific CAR T cells with CD28 co-stimulus produced similar levels of cytokines upon stimulation in vitro, anti-tumor activity in vivo differed. Since spacer length was the only difference in design, the reduction in L1CAM-LS-CD28/ζ in vivo performance can clearly be attributed to the long spacer element. Spacer elements, when derived from IgG molecules, tend to bind FcγR^+^ cells, which can cause activation-induced cell death in CAR T cells. We used a modified long spacer element derived from IgG4, carrying two substituting mutations (L235D and N297Q) within the CH2 domain demonstrated to prevent activation-induced cell death by FcγR^+^ binding [18]. Nevertheless, we observed apoptosis in significantly more human L1CAM-LS-CD28/ζ CAR T cells after in vitro stimulation with L1CAM-positive tumor cells compared to the short spacer design. Together with our observation that this L1CAM-specific CAR subgroup showed significantly higher in vitro cytotoxicity compared to L1CAM-SS-CD28/ζ CAR T cells, we hypothesize that antigen-driven overactivation after repeated antigen encounter is accountable for the reduced in vivo survival and anti-tumor activity of L1CAM-LS-CD28/ζ CAR T cells. This hypothesis is supported by our previous report demonstrating that L1CAM-specific CAR T cells with long spacer element are more susceptible to Fas/FasL-induced fratricide [16].

Our CAR designs incorporated components derived from human sequences, including 4-1BB and CD28 co-stimulatory domains. This might be of concern as homology between mouse and human sequences for CD28 (85%) and 4-1BB (59%) endodomains differs. Although we cannot exclude that human derivation causes impaired 4-1BB signaling in murine T cells, reducing efficacy of the 4-1BB CAR design, there are two arguments against this. TRAF binding motifs in the 4-1BB cytoplasmic tails are 100% conserved between mouse and human [33], supporting preservation of signaling cascade initiation. Murine HER2-SS-4-1BB/ζ CAR T cell ability to eradicate solid tumors supports that the human-derived 4-1BB domain functioned properly in murine T cells.

Second-generation CD28-based CAR T cells are prone to exhaustion after repeated antigen exposure, resulting in T cell dysfunction and lack of persistence [34,35,36,37]. We also observed upregulation of PD1 and TIM3 immune checkpoint molecules, indicative for exhaustion, on tumor-infiltrating L1CAM-specific CAR T cells with CD28 signaling. This exhaustion might account for the transient character of anti-tumor control in our model, as tumors in all mice eventually relapsed. The lack of detectable PD1 and TIM3 on CAR T cells utilizing 4-1BB co-stimulation can be interpreted as a lack of CAR T cell activation, since both surface markers are also transiently upregulated by T cell activation [38,39]. Strategies combining CAR T cell therapy with checkpoint blockade have been investigated with promising results [24,40]. Interestingly, Guedan et al. recently demonstrated that substitution of a single amino acid in the CD28 co-stimulatory domain in mesothelin-targeting CAR T cells promoted anti-tumor control and reduced exhaustion and terminal differentiation in the CAR T cells [41]. Similarly, Feucht et al. produced CD19-specific CAR T cells with enhanced therapeutic profiles by removing the two C-terminally located ITAM motifs in the CD28 co-stimulatory domain [42]. These examples indicate that additional approaches can be utilized to prolong functionality of second-generation CAR T cells utilizing CD28 signaling without compromising their potency.

## 5. Conclusions

Here we show that CAR T cells targeting L1CAM with CD28 signaling more effectively control tumor growth than CAR T cell designs using 4-1BB signaling. The superiority of CAR designs employing CD28 signaling was also demonstrated with HER2-targeting CAR T cells, but only for the design using a long spacer element. We also present a new mouse model for robust preclinical CAR evaluation supporting evaluation of CAR T cell persistence, trafficking and effector function. Using mono-specific murine instead of polyclonal human T cells provides an effective means to investigate CAR T cell action under physiological conditions and excludes confounding factors due to species-specific mismatches. Our robust preclinical evaluation of second-generation CAR T cell designs indicate that CD28 signaling may produce more potent candidates to treat solid tumors because they more effectively migrate to and expand at the tumor site.

## Data Availability

Data is presented within the article and supplementary material.

## Supplementary Materials

The following are available online at https://www.mdpi.com/2072-6694/13/5/1050/s1, Figure S1: Transduction efficacy of CAR constructs, Figure S2: Target antigen surface expression on tumor cells, Figure S3: Flow cytometry gating strategy to identify T cells within co-cultures with tumors cells, Figure S4: PDX mouse model treated with single dose of 1 × 10^6^ human L1CAM-specific CAR T cells.
