# Supplementary material for: CD28 Co-Stimulus Achieves Superior CAR T Cell Effector Function against Solid Tumors Than 4-1BB Co-Stimulus
Source: Cancers (Basel). 2021 Mar 2;13(5):1050. doi: 10.3390/cancers13051050 (PMC7958604; doi:10.3390/cancers13051050)
Supplement: Supplementary file 1 [file cancers-13-01050-s001.pdf]

cancers

Article

CD28 Co-Stimulus Achieves Superior CAR T Cell Effector Function against Solid Tumors Than 4-1BB Co-Stimulus

Ana Textor, Laura Grunewald, Kathleen Anders, Anika Klaus, Silke Schwiebert, Annika Winkler, Maria Stecklum, Jana Rolff, Anton G. Henssen, Uta E. Höpken, Angelika Eggert, Johannes H. Schulte, Michael C. Jensen, Thomas Blankenstein and Annette Künkele

Citation: Textor, A.; Grunewald, L.; Anders, K.; Klaus, A.; Schwiebert, S.; Winkler, A.; Stecklum, M.; Rolff, J.; Henssen, A.G.; Höpken, U.E.; et al. CD28 Co-Stimulus Achieves Superior CAR T Cell Effector Function against Solid Tumors Than 4-1BB Co-Stimulus. *Cancers* **2021**, *13*, 1050. https://doi.org/10.3390/cancers13051050

Academic Editor: Anna Pasetto

Received: 1 February 2021
Accepted: 24 February 2021
Published: 2 March 2021

Publisher's Note: MDPI stays neutral with regard to jurisdictional claims in published maps and institutional affiliations.

Copyright: © 2021 by the authors. Licensee MDPI, Basel, Switzerland. This article is an open access article distributed under the terms and conditions of the Creative Commons Attribution (CC BY) license (http://creativecommons.org/licenses/by/4.0/).

Supplementary Materials:

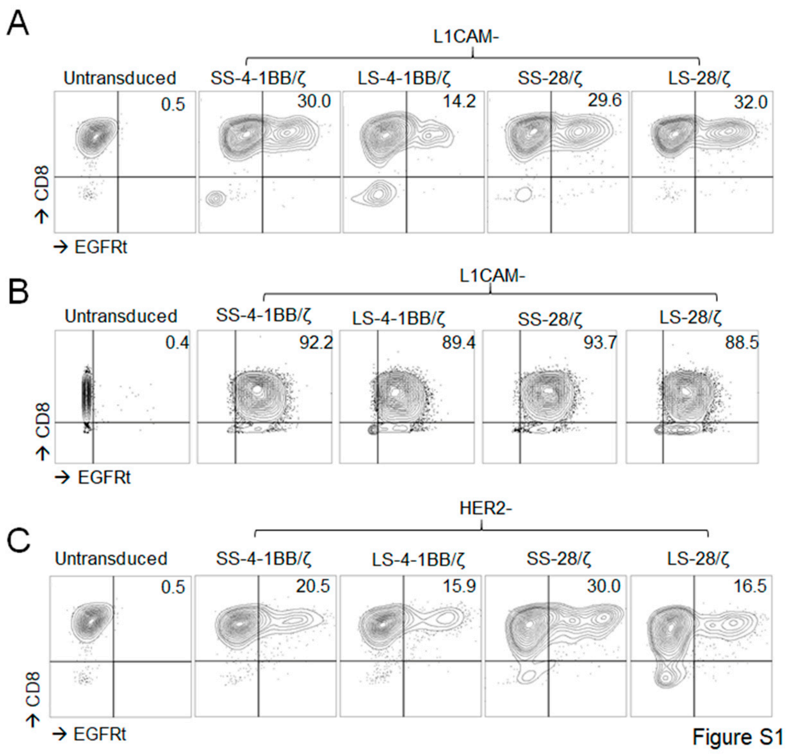

Figure S1. Transduction efficacy of CAR constructs. (**A**) Mouse splenocytes derived from ChRLuc/OT-1/Rag$^{-/-}$ mice were transduced with retroviruses encoding L1CAM-specific SS-4-1BB/ζ, LS-4-1BB/ζ, SS-28/ζ or LS-28/ζ CARs. Cells were stained with cetuximab (anti-EGFR antibody) and anti-CD8 antibody to assess transduction efficacy by flow cytometry. Untransduced CD8^{+} T cells served as negative control. (**B**) Representative flow cytometry plots showing CD8 and EGFRt expression on CD8^{+} human T cells transduced with L1CAM-specific SS-4-1BB/ζ, LS-4-1BB/ζ, SS-28/ζ or LS-28/ζ constructs after MACS enrichment. Untransduced T cells served as negative control. (**C**) Mouse splenocytes derived from ChRLuc/OT-1/Rag$^{-/-}$ mice were transduced with retroviruses encoding HER2-SS-4-1BB/ζ, HER2-LS-4-1BB/ζ, HER2-SS-28/ζ or HER2-LS-28/ζ CARs and stained with cetuximab (anti-EGFR antibody) and anti-CD8 antibody to determine transduction efficacy by flow cytometry.

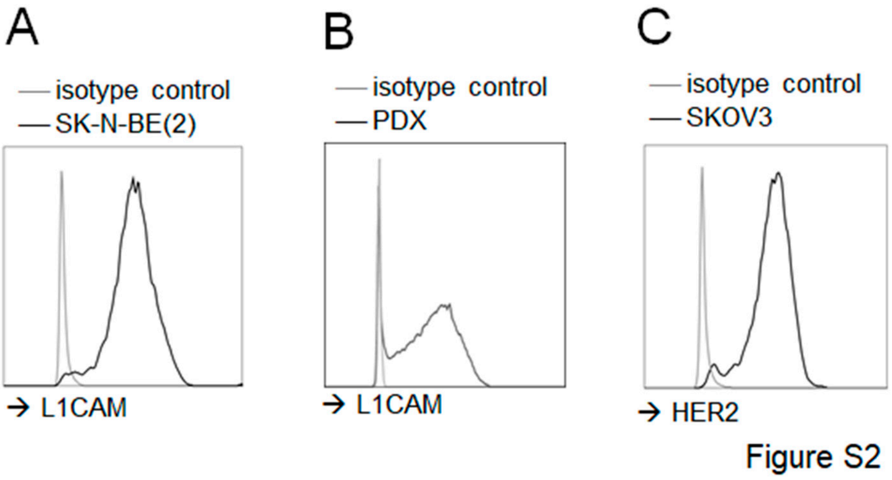

Figure S2. Target antigen surface expression on tumor cells. Flow cytometry analysis of L1CAM expression on SK-N-BE(2) neuroblastoma cells (**A**) and single cells prepared from a PDX stained with anti-L1CAM monoclonal antibody (**B**). (**C**) Flow cytometry analysis of HER2 expression on SKOV3 ovarian carcinoma cells stained with anti-HER2 antibody. Cells stained with the isotype control antibody served as control.

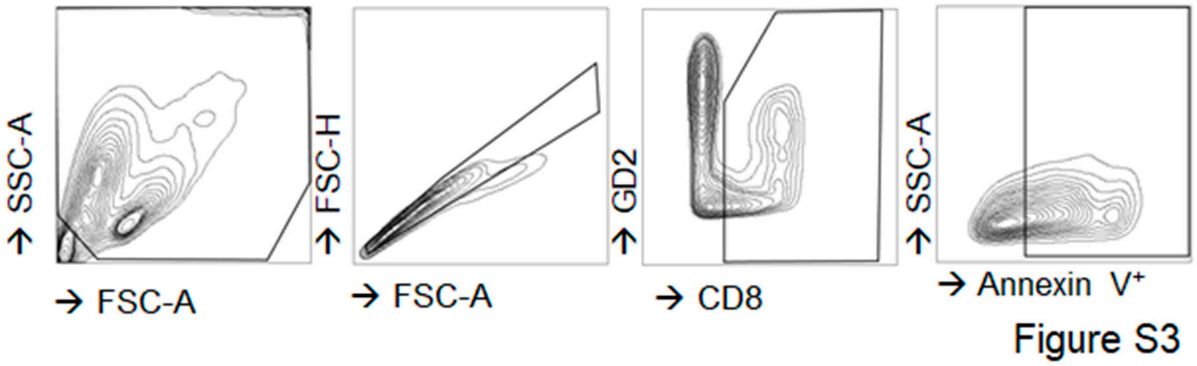

Figure S3. Flow cytometry gating strategy to identify T cells within co-cultures with tumors cells. Single cell suspension was stained with anti-GD2 (to discriminate from GD2^{+} tumor cells), anti-CD8 and annexin V conjugated with different fluorochromes and analyzed by flow cytometry. Among single cells, T cells (CD8^{+}, GD2^{-}) were selected to assess annexin V positivity.

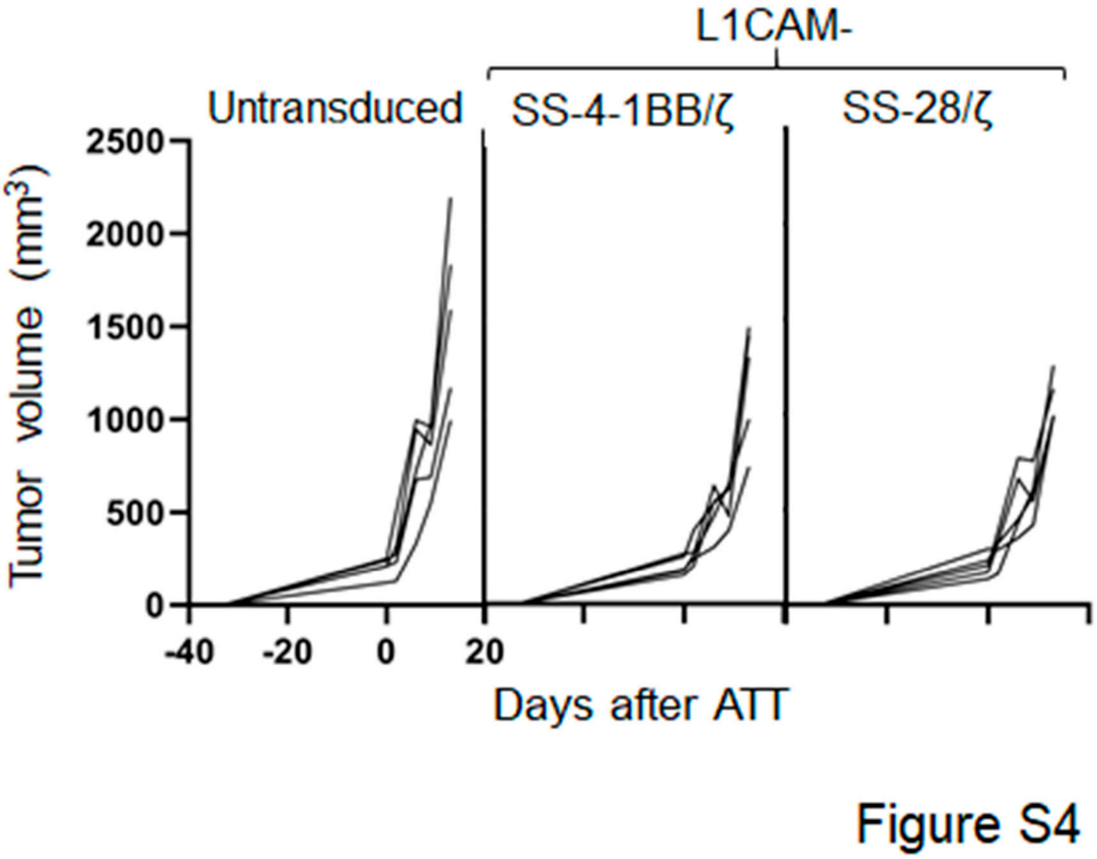

Figure S4. PDX mouse model treated with single dose of 1x10[6] human L1CAM-specific CAR T cells. NOG mice were engrafted with a neuroblastoma PDX and treated 40 days later with a single dose of 1x10[6] human L1CAM-SS-28/ζ (n=5), L1CAM-SS-28/ζ (n=5) or untransduced T cells (n=5).
